# Redesign of Anode Catalyst for Sustainable Survival of Fuel Cells

**DOI:** 10.1002/advs.202307073

**Published:** 2024-01-15

**Authors:** Keonwoo Ko, Dongsu Kim, Jiho Min, Bathinapatla Sravani, Yunjin Kim, Sanghyeok Lee, Taejun Sul, Segeun Jang, Namgee Jung

**Affiliations:** ^1^ Graduate School of Energy Science and Technology (GEST) Chungnam National University 99 Daehak‐ro, Yuseong‐gu Daejeon 34134 Republic of Korea; ^2^ School of Mechanical Engineering Kookmin University Seoul 02707 Republic of Korea; ^3^ Fuel Cell Laboratory Korea Institute of Energy Research (KIER) 152 Gajeong‐ro, Yuseong‐gu Daejeon 34129 Republic of Korea

**Keywords:** polymer electrolyte membrane fuel cells, anode catalysts, multifunctional carbon layer, fuel starvation, shut‐down/start‐up, open circuit voltage holding

## Abstract

Polymer electrolyte membrane fuel cells (PEMFCs) suffer from severe performance degradation when operating under harsh conditions such as fuel starvation, shut‐down/start‐up, and open circuit voltage. A fundamental solution to these technical issues requires an integrated approach rather than condition‐specific solutions. In this study, an anode catalyst based on Pt nanoparticles encapsulated in a multifunctional carbon layer (MCL), acting as a molecular sieve layer and protective layer is designed. The MCL enabled selective hydrogen oxidation reaction on the surface of the Pt nanoparticles while preventing their dissolution and agglomeration. Thus, the structural deterioration of a membrane electrode assembly can be effectively suppressed under various harsh operating conditions. The results demonstrated that redesigning the anode catalyst structure can serve as a promising strategy to maximize the service life of the current PEMFC system.

## Graphical abstract

In this study, an anode catalyst based on Pt nanoparticles encapsulated in a multifunctional carbon layer (MCL) is redesigned, acting as a molecular sieve layer and protective layer. Electrochemical studies have shown that MCL helped suppress the structural deterioration of a membrane electrode assembly and maximize the service life of the PEMFC system.

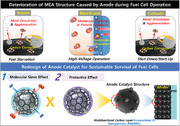

## 1 Introduction

For decades, the research on catalysts for polymer electrolyte membrane fuel cells (PEMFCs) have been focused on the cathode, where the oxygen reduction reaction (ORR) occurs.^[^ ^1^, ^2^, ^3^, ^4^, ^5^, ^6^, ^7^, ^8^, ^9^, ^10^, ^11^ ^]^ As the performance of a PEMFC system is primarily governed by the ORR activity of a cathode catalyst,^[^ ^1^, ^2^, ^3^, ^4^, ^5^, ^6^, ^7^, ^8^, ^9^, ^10^, ^11^ ^]^ considerable research efforts have been devoted to prevent the deterioration of the cathode catalyst to prolong the durability of the PEMFC system.^[^ ^8^, ^9^, ^10^, ^11^ ^]^ However, the development of the anode catalyst has been relatively neglected for promoting the hydrogen oxidation reaction (HOR) with a fast reaction rate as it poses a small contribution to fuel cell performance compared to the cathode catalyst.^[^ ^12^ ^–^ ^14^ ^]^

**Scheme 1:**
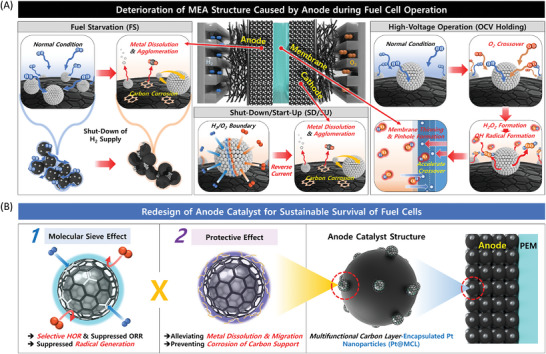
Schematics of A) the systemic issues caused by the anode in PEMFC under real‐world operating conditions and B) the effects of MCL‐coated anode catalysts.

However, from a perspective of practical applications, the performance of fuel cell systems starts to severely deteriorate in real‐world conditions owing to the transient phenomena generated at the anode, as illustrated in **Scheme** **1**A.^[^ ^15^, ^16^, ^17^, ^18^, ^19^, ^20^, ^21^, ^22^, ^23^ ^]^ First, the anode catalyst and carbon support are considerably degraded within a short period owing to the reverse potential created by the H_2_ fuel deficiency under an abnormal condition (fuel starvation, FS) when the fuel cell is restarted.^[^ ^15^, ^16^, ^17^ ^]^ Second, reactive OH∙ radicals are formed at the anode because of the O_2_ crossover from the cathode during high‐voltage operation, resulting in membrane thinning, and pinhole formation.^[^ ^18^, ^19^, ^20^ ^]^ Third, under shut‐down/start‐up (SD/SU) conditions, the reverse current generated by the H_2_/O_2_ boundary formation at the anode severely deteriorates the cathode carbon support and catalyst.^[^ ^21^, ^22^, ^23^ ^]^ Therefore, the structural deterioration of a membrane electrode assembly (MEA) due to the abnormal conditions at the anode significantly affects the service life of fuel cells.

Although these technical issues have been raised over the past few decades, researchers have focused on providing individual solutions to address these limitations, such as the addition of functional materials in electrodes,^[^ ^15^, ^21^, ^24^, ^25^, ^26^, ^27^, ^28^, ^29^, ^30^ ^]^ reinforcement of membrane structure,^[^ ^31^, ^32^, ^33^, ^34^, ^35^ ^]^, and the introduction of additional balance of plant facilities.^[^ ^21^, ^25^, ^36^ ^]^ However, these approaches increase the cost and volume of the fuel cell system. To the best of our knowledge, no holistic ideas have been reported to solve the aforementioned problems, possibly because of undermining the insight that the design of the anode catalyst can serve as a point of breakthrough to solve these systemic issues.

In this study, we devise an interesting approach to address the aforementioned issues of performance degradation during fuel cell operation. We focus on redesigning the anode catalyst structure to develop new avenues of performance improvement. As illustrated in Scheme 1B, we encapsulate Pt nanoparticles in a multifunctional carbon layer (MCL), which is an ultrathin, porous carbon layer that enables selective HOR of Pt nanoparticles via molecular sieve effect. Simultaneously, it serves as a protective layer to prevent the dissolution and agglomeration of Pt particles even under abnormal operating conditions. The MCL‐coated Pt nanoparticles (Pt@MCL) aim to alleviate the systemic problems caused by the anode under FS, SD/SU conditions, and high‐voltage operations, which are simulated by an open circuit voltage (OCV) holding test. As a result, it is demonstrated through accelerated stress tests (ASTs) under each condition that the unified material design is effective in maximizing the service life of PEMFC systems.

## 2 Results and Discussion

### 2.1 HOR Selectivity and Protective Effect Induced by MCL

**Figure 1:**
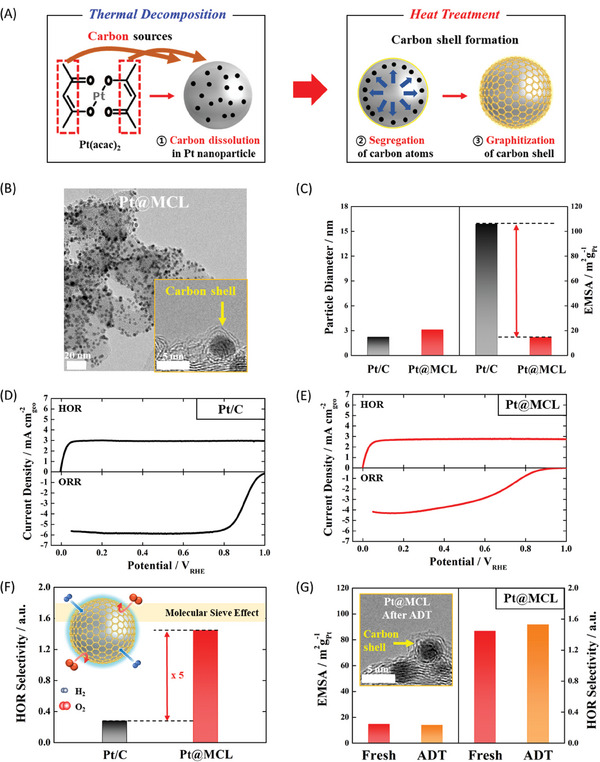
A) Schematic of the formation of MCL on Pt nanoparticles. B) TEM image of Pt@MCL catalyst. Inset: HR‐TEM image of MCL formed on the surface of a Pt nanoparticle. C) Correlation between the average particle diameter and EMSA of the catalysts. HOR and ORR polarization curves of D) Pt/C, and E) Pt@MCL catalysts. F) Comparison of HOR selectivity. G) Variations in electrochemical properties of Pt@MCL catalyst before and after ADT. Insets: the HR‐TEM image of Pt@MCL catalyst after ADT.

The Pt@MCL catalyst was prepared through a solvothermal reaction aimed at decomposing Pt acetylacetonate (Pt(acac)_2_) serving as a precursor. Subsequently, we performed a high‐temperature heat treatment in Ar atmosphere. During the synthesis process at 300 °C, the acetylacetonates generated a trace amount of carbon sources, which were absorbed into the lattice of Pt nanoparticles. This absorption enabled the formation of MCL on the surface of Pt nanoparticles through carbonization during the subsequent heat treatment (refer to **Figure** **1**A).^[^ ^37^, ^38^, ^39^, ^40^, ^41^, ^42^, ^43^ ^]^ As observed from the transmission electron microscopy (TEM) image depicted in Figure 1B,C well‐dispersed Pt nanoparticles (average size: ≈3 nm, Figure 1C) were formed on the carbon support (Figure S1, Supporting Information). Moreover, the high‐resolution (HR)‐TEM image conclusively confirmed the presence of the ultrathin MCL formed on the Pt surface (inset in Figure 1B). The thickness of the MCL is identified to be <0.6 nm, implying the formation of a 1 to 1.5 layer (Figure S2, Supporting Information).^[^ ^44^, ^45^, ^46^ ^]^ In addition, as shown in Figure S3 (Supporting Information), the N1s XPS spectrum for the Pt@MCL catalyst indicates that the MCL is a pristine carbon layer because the carbon source of the Pt(acac)_2_ precursor purely forms that layer without additional N‐doping treatment. Thus, we can infer that even after heat treatment at 800 °C, the Pt nanoparticles of the Pt@MCL catalyst maintained their small particle size because of the encapsulation provided by the MCL.

In comparison, the commercial Pt/C catalyst exhibited a particle size of ≈2.7 nm, slightly smaller than that of the Pt@MCL catalyst (Figures S1 and S4, Supporting Information). However, the presence of the overcoated MCL significantly contributed to the substantially lower exposed metal surface area (EMSA) of the Pt@MCL catalyst compared to that of Pt/C. This observation was supported by the CO stripping curves and CVs (Figure S5, Supporting Information). The pronounced influence of MCL, with its appropriate carbon pores (defects), on the electrochemical properties of the metal nanoparticles is exemplified in Figure 1C. The selectivity of the HOR exhibited by the anode catalyst presumes a pivotal role in addressing the aforementioned challenges encountered during the operation of PEMFCs. In this context, the HOR selectivity of the Pt@MCL and Pt/C catalysts underwent analysis by comparing their HOR activity with their ORR activity (refer to Figure 1D,E). Consequently, the Pt@MCL catalyst demonstrated HOR activity comparable to that of Pt/C, whereas the ORR performance was significantly suppressed in comparison to the Pt/C catalyst. This outcome can be explained based on the molecular sieve effect induced by the porous MCL, which facilitated the permeation and selective reaction of H_2_ gas on the surface of the Pt nanoparticles, characterized by its relatively small kinetic diameter. Conversely, the diffusion of O_2_ through the MCL was effectively impeded owing to its larger kinetic diameter.^[^ ^47^, ^48^ ^]^ Consequently, the Pt@MCL catalyst exhibited a superior HOR selectivity (≈5 times) than the Pt/C catalyst (Figure 1F).

Moreover, the stability and reliability of the MCL in the Pt@MCL catalyst were verified through TEM analysis (Figure S6, Supporting Information) and electrochemical analyses (Figure S7, Supporting Information) following an accelerated degradation test (ADT) involving 10000 potential cycles. Even under harsh ADT conditions, the size and shape of the Pt nanoparticles remained unchanged, and the MCL persisted on the Pt surface (Figure S6, Supporting Information). Notably, the EMSA and HOR selectivity experienced minimal fluctuations before and after the ADT (Figure 1G), implying the protective role of MCL. Thus, the MCL on the Pt surface can be expected to simultaneously function as a molecular sieve layer and a protective layer in the anode of PEMFCs.

### 2.2 Enhanced MEA Durability under Fuel Starvation Conditions

**Figure 2:**
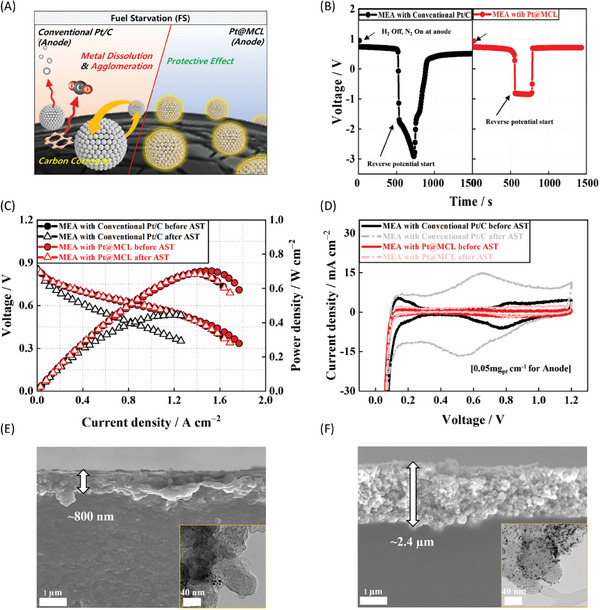
A) Schematic of degradation mechanism of anode catalyst under FS conditions. B) Time‐dependent cell potential behaviors of MEAs with conventional Pt/C and Pt@MCL electrodes under accelerated simulation conditions for FS by switching H_2_ to N_2_ supply at the anode with constant current density of 0.2 A cm^−2^. C) Polarization curves and D) CVs with MEAs before and after the accelerated FS tests. Cross‐sectional SEM and TEM (inset) images of the anodes of the MEAs with E) conventional Pt/C, and F) Pt@MCL after the test.

In **Figure** **2**A, under FS conditions, unwanted reactions such as the oxygen evolution reaction (OER, 2*H*_2_ *O* → *O*_2_ + 4*H* ^+^ + 4*e* ^−^, *E* ^0^ =  1.23 V vs RHE), and carbon oxidation reaction (COR, *C* + 2*H*_2_ *O* → *CO*_2_ + 4*H* ^+^ + 4*e* ^−^,  *E* ^0^ =  0.21 V vs RHE) occur at the anode to compensate for the electron deficiency and maintain redox balance with the cathode. However, due to slow kinetics, the COR currents were typically observed at potentials higher than 1.4 V.^[^ ^15^, ^49^ ^]^ Consequently, during H_2_ fuel depletion, anodic currents were initially generated by OER, followed by COR rather than the HOR, resulting in severe carbon corrosion, significant Pt dissolution, and agglomeration.^[^ ^16^, ^50^, ^51^ ^]^

The time‐dependent cell potential behavior of MEAs with conventional Pt/C and Pt@MCL electrodes under accelerated FS simulation conditions is portrayed in Figure 2B. The accelerated FS tests involved replacing the H_2_ fuel to N_2_ at the anode while maintaining a constant current density of 0.2 A cm^−2^. The cell voltage remained stable until H_2_ was completely depleted (≈600 s), after that the OER and COR currents induced a reverse potential that was sustained for 120 s to observe the degradations in the anode catalyst. The MEA with conventional Pt/C rapidly transitioned through the reverse potential range dominated by OER (−0.8 to −1.1 V) and reached COR potential ranges below −1.7 V, resulting a sharp decline in cell voltage to −2.9 V.^[^ ^15^ ^]^ Therefore, the OER activity of the Pt/C electrode deteriorated rapidly because of the Pt nanoparticle dissolution, consequently triggering carbon corrosion (COR) as a compensatory anodic current. Interestingly, the MEA with the Pt@MCL catalyst exhibited a plateau curve at −1.0 V for 120 s, suggesting that the Pt@MCL electrode could sustain its OER activity without corrosion of carbon supports for a relatively extended period during the reverse potential regime, owing to the protective effect of the MCL on the Pt nanoparticles.^[^ ^40^, ^42^ ^]^

The *I–V* curves obtained before and after the accelerated FS test are plotted in Figure 2C. Both the Pt/C and Pt@MCL MEAs exhibited similar a BOL performance. However, after exposure to the reverse potential during the FS test, the MEA with Pt@MCL demonstrated a 53 % higher peak power density (PPD) (0.687 W cm^−2^) compared to the conventional Pt/C MEA (0.449 W cm^−2^). For the conventional MEA, the charge–transfer resistance (R_ct_) increased significantly at a high voltage of 0.8 V after the FS test, with a predominating activation overpotential (Figure S8, Supporting Information). Simultaneously, the electrochemically active surface area (ECSA) of the anode, which was evaluated from CV (Figure 2D), decreased by ≈84.5 % (from 58.94 to 9.14 m^2^ g_Pt_^−1^). Notably, prominent hydroquinone–quinone redox peaks emerged within the potential range of 0.5–0.7 V, indicative of carbon support surface oxidation by COR at the anode.^[^ ^49^, ^52^, ^53^, ^54^, ^55^ ^]^ Conversely, the CV of the Pt@MCL electrode experienced minimal variations even after the FS test, while exhibiting a significantly smaller hydrogen underpotential deposition (H_upd_) area.

As illustrated in Figure 2E,F, the change in the anode CVs of the MEAs could be elucidated by the changes in anode thickness (scanning electron microscopy (SEM) images) and catalyst structure (inset in TEM images) during the accelerated FS tests. When compared to the initial MEAs (Figure S9, Supporting Information), the anode thickness of the conventional MEA with Pt/C decreased dramatically to 0.8 µm after the FS test, whereas that of the MEA with Pt@MCL barely varied. Furthermore, for the Pt@MCL electrode, the Pt nanoparticles were well‐preserved on the carbon support, whereas the majority of the Pt nanoparticles were degraded due to metal dissolution by the applied reverse potentials for the conventional Pt/C electrode. As such, the reverse potential behavior during the FS was significantly influenced by the retention period of OER, which could delay the onset of severe COR.^[^ ^50^, ^53^, ^56^, ^57^ ^]^ Accordingly, it was believed that the Pt@MCL electrode maintained a constant OER potential with the corresponding anodic current since the MCL could effectively suppress the Pt dissolution and migration even at high potentials during the OER under the FS conditions. To confirm this, an additional experiment was performed, extending the exposure time of the reverse potential to the MEA with Pt@MCL to 1800 s. In Figure S10A (Supporting Information), the MEA with Pt@MCL indicated a plateau curve at −1.0 V for a long duration of 1040 s, after that the cell voltage rapidly declined to −2.3 V. Consequently, the performance degradation (Figure S10B, Supporting Information) and the carbon corrosion (Figure S10C, Supporting Information) were observed. Therefore, the MCL on the Pt nanoparticles played a crucial role in alleviating the COR during the FS test by extending the duration of OER and thereby enhancing the durability of the MEA with Pt@MCL against FS.

### 2.3 Enhanced MEA Durability under SD/SU Conditions

**Figure 3:**
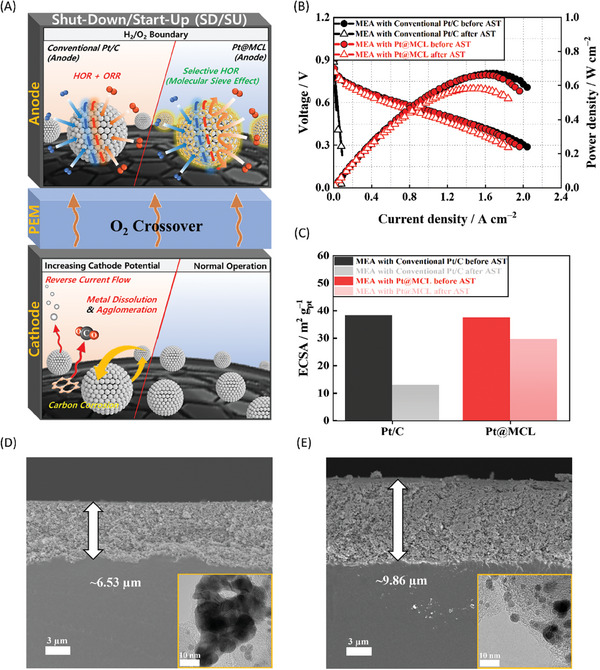
A) Schematic of cathode degradation under SD/SU condition. B) Polarization curves before and after accelerated SD/SU test. C) Calculated ECSA before and after SD/SU test. D–E) Cross‐sectional SEM images (cathode) and TEM images (catalyst) of D) conventional MEA with Pt/C, and E) MEA with Pt@MCL.

When the fuel cells were subjected to repetitive SD/SU conditions, especially for automotive applications, and the H_2_/O_2_ boundary could form locally in the anode owing to the O_2_ crossover from the cathode through the membrane (**Figure** **3**A). Under these conditions, undesirable ORR occurred at the anode, and resulting in reverse current flow that lowered the local electrolyte potential (e.g., from 0 to −0.6 V).^[^ ^21^, ^22^, ^37^, ^58^, ^59^ ^]^ Consequently, the reverse current anomalously increased the cathode potential beyond 1.4 V, causing severe carbon support corrosion at the cathode. Prior research has extensively applied high‐crystalline carbon materials as the cathode support to improve the MEA durability under the SD/SU conditions.^[^ ^60^, ^61^, ^62^ ^]^ However, for high‐crystalline carbon supports with high stability, securing an adequately high surface area to immobilize Pt nanoparticles on the surface became challenging.

In this study, we proposed a design for the anode catalyst that could fundamentally prevent ORR at the anode by incorporating a porous MCL onto Pt nanoparticles. The MCL induced a molecular sieve effect, selectively facilitating permeation of H_2_ gas while impeding the diffusion of O_2_, which had a relatively larger kinetic diameter than H_2_. This design minimized the generation of reverse current. To validate the molecular sieve effect of the MCL and assess the durability of the MEA, we conducted an accelerated SD/SU test using a protocol that simulated the formation of an H_2_/O_2_ boundary by simultaneously supplying H_2_ and air to the anode.^[^ ^63^, ^64^, ^65^, ^66^ ^]^ The detailed experimental procedure is described in the Experimental Section.

The current–voltage (*I–V*) curves of the MEAs with Pt/C and Pt@MCL as anode catalysts before and after the accelerated SD/SU test are plotted in Figure 3B. Although both MEAs shared the same Pt/C cathode, the conventional MEA exhibited significant degradation in single‐cell performance compared to the MEA with Pt@MCL following the SD/SU test. The MEA with Pt@MCL demonstrated a 82.73 % reduction in charge–transfer resistance (*R*_ct_) (1.930 Ω cm^2^) at 0.8 V compared to the conventional MEA (11.176 Ω cm^2^) (Figure S11, Supporting Information). Although the ECSA of the MEA with Pt@MCL well maintained, the conventional MEA experienced a severe reduction in ECSA (11.79 m^2^ g_Pt_^−1^), reduced by 69.16 %. The decline in ECSA for the cathode of the conventional MEA was primarily caused by the detachment and agglomeration of the Pt nanoparticles under carbon corrosion during the SD/SU test.^[^ ^23^, ^67^, ^68^ ^]^ The SEM image of the conventional MEA post‐SD/SU test is illustrated in Figure 3D, exhibiting a noticeable decrease in the thickness of the cathode catalyst layer (CL) compared to the fresh MEA (≈12.2 µm, Figure S12, Supporting Information), along with noticeable Pt agglomeration. In contrast, the MEA with Pt@MCL exhibited negligible structural variations in the cathode CL (Figure 3E). These results led to the conclusion that introducing Pt@MCL with a molecular sieve effect effectively prevented reverse current flow and carbon corrosion at the cathode under SD/SU conditions.

### 2.4 Enhanced MEA Durability under High‐Voltage Operating (OCV Holding) Conditions

**Figure 4:**
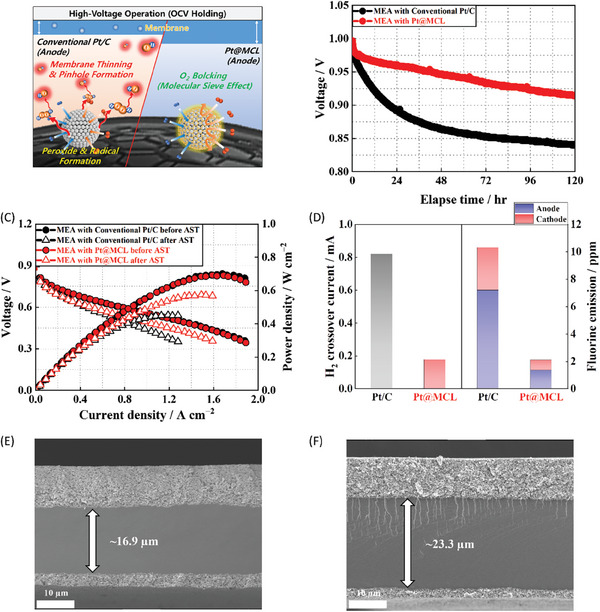
A) Schematic of chemical degradation of MEA by radical attack. B) OCV decay curves over 120 h under accelerated OCV holding test conditions. C) Polarization curves before and after OCV holding test. D) H_2_ crossover current and fluoride‐ion emission after OCV holding test. Cross‐sectional SEM images of E) conventional MEA with Pt/C, and F) MEA with Pt@MCL after OCV holding test.

Specifically, in automotive fuel cells, the fuel cell system operates in an idle state for over 30 % of its operating time under typical driving conditions.^[^ ^69^, ^70^ ^]^ In this state, the fuel cell system experiences a high cell potential (>0.8 V, approximate to OCV), and O_2_ gas molecules tend to diffuse through the membrane from the cathode to the anode. Consequently, a direct combustion reaction between H_2_ and O_2_ occurs, leading to the formation of hydrogen peroxide (H_2_O_2_).^[^ ^18^, ^71^, ^72^, ^73^, ^74^ ^]^ Furthermore, this reaction generates highly reactive hydroxyl (HO∙) and hydroperoxyl (HOO∙) radicals through the Fenton reaction between H_2_O_2_ and cations such as Fe^2+^, which are released from bipolar plates and/or humidifiers.^[^ ^34^, ^71^, ^75^ ^]^ These radicals induce decomposition of the main and side chains of the membrane, resulting in membrane thinning, pinhole formation, and ultimately, a shortened fuel cell lifetime. Additionally, they contribute to performance degradation due to limited proton transport in the electrode (**Figure** **4**A).

Under the conditions of an OCV holding test with a relatively high‐temperature of 90 °C and low RH of 30 %, the chemical degradation of the membrane and ionomer induced by radicals was accelerated.^[^ ^34^, ^64^, ^76^ ^]^ Therefore, we conducted an OCV holding test to evaluate the molecular sieve effect of the MCL on the chemical stability of the prepared MEAs. Figure 4B depicts the decay curves of OCV for the MEAs with Pt/C and Pt@MCL over 120 h during the accelerated OCV holding test, where O_2_ gas was directly supplied to the cathode instead of air. Consequently, the MEA with Pt@MCL exhibited a significantly lower decay rate of 0.683 mV h^−1^ compared to the conventional MEA (1.275 mV h^−1^), indicating a substantial suppression of the direct combustion reaction between H_2_ and O_2_ for H_2_O_2_ formation at the anode of the MEA with Pt@MCL. The improved OCV stability can be attributed to the molecular sieve effect of the MCL, effectively blocking the direct access of O_2_ to the Pt surface while selectively permitting H_2_ permeation for the HOR. After the test, the PPD of the MEA with Pt@MCL was much higher at 0.575 W cm^−2^ compared to that of the conventional MEA at 0.455 W cm^−2^, as presented in Figure 4C.

The alleviated chemical degradation of the MEA with Pt@MCL was further confirmed by 75 and 84 % lower H_2_ crossover current and fluoride‐ion emission, respectively, compared to the conventional MEA (Figure 4D). The detailed time‐dependent fluoride‐ion emission rates measured every 24 h are shown in Figure S13 (Supporting Information).These findings signified that the selective HOR could effectively prevent H_2_O_2_ formation at the anode while minimizing degradation of the fluorine‐containing perfluorosulfonic acid membrane and ionomer, especially on the anode side. Evidently, the conventional MEA exhibited notable membrane thinning, with a reduced thickness of 16.9 µm from the initial value of 25 µm (Figure S14, Supporting Information), whereas the MEA with Pt@MCL retained its original thickness (Figure 4E,F).^[^ ^77^, ^78^ ^]^ In case of the anode catalysts, severe Pt agglomeration was observed only in the conventional MEA (Figure S15, Supporting Information). Owing to the combined effect of ionomer degradation and Pt agglomeration, the charge–transfer resistance (R_ct_) of the conventional MEA increased significantly at 0.8 V, as depicted in Figure S16 (Supporting Information). Accordingly, the molecular sieve effect induced by the MCL contributed toward improving the chemical stability of the MEA by mitigating radical generation.

## 3 Conclusion

This study developed a novel approach to address systemic issues encountered during the operation of PEMFCs by designing MCL‐encapsulated Pt nanoparticles as a promising anode catalyst, diverging from previous studies that primarily focused on the cathode for ORR. An ultrathin, porous MCL was synthesized on the Pt nanoparticle surface through a solvothermal reaction, followed by high‐temperature heat treatment utilizing a trace amount of carbon sources present in the Pt precursor. The resulting MCL served as a molecular sieve layer and protective barrier, enabling selective HOR while preventing Pt nanoparticle dissolution, and agglomeration under abnormal operating conditions. The results demonstrated the effectiveness of the molecular sieve effect and protective properties of the MCL for prolonging the service life of the PEMFC systems. **Table** **1** summarized the effects of the MCL on the overall durability of the MEA and the detailed each degradation rate is shown in Table S1 (Supporting Information). Notably, the structural degradation of the MEA, including the CL, membrane, and ionomer, was mitigated by alleviating abnormal phenomena such as carbon corrosion, hydrogen peroxide (H_2_O_2_) formation, and radical generation occurring during FS, SD/SU, and OCV conditions. Overall, this research provides valuable insights into the design of anode catalysts based on MCL‐encapsulated Pt nanoparticles with unique characteristics (molecular sieve effect and protective properties), emphasizing the significance of considering the anode catalyst structure, and that has been overlooked thus far, as a potential breakthrough for addressing the issues in PEMFC systems.

**Table 1:** Summary of the effects of Pt@MCL at anode on MEA durability.

Target Materials	Anode CL	Cathode CL	Membrane
Simulated AST	Fuel starvation	SD/SU	OCV holding
Main Role of MCL	Protective layer	Molecular sieve	Molecular sieve
Effects of MCL	Elongated duration of OER and suppressed COR	Suppressed reverse current at H_2_/O_2_ boundary	Suppressed radical generation on Pt surface

## 4 Experimental Section

### Chemicals and Materials

Carbon black (Vulcan XC‐72, USA) was purchased from Cabot Inc. (Alpharetta, GA, and USA). 1‐Octadecene (90 %) was acquired from Alfa‐Aesar (USA). Platinum acetylacetonate (Pt(acac)_2_, 97 %), oleylamine (70 %), Nafion ionomer (5 wt. %), and 2‐propanol (99.5 %) were procured from Sigma–Aldrich Inc. (St. Louis, MO, and USA). n‐Hexane (95 %), ethanol (95 %), isopropyl alcohol (99.5 %), and dipropylene glycol (97 %) were acquired from Samchun Pure Chemical (Daejeon, South Korea). A rotating disk electrode (RDE) with a glassy carbon (geometric area: 0.196 cm^2^) was purchased from Metrohm‐Autolab (Netherlands). Nafion membrane (NRE 211) was acquired from Dupont Inc (USA). Gas diffusion layers (GDLs) (Sigracet 39BB) were procured SGL Carbon Inc (Germany). Teflon gaskets was purchased from CNL Energy (South Korea). A commercial 20 wt. % Pt/C catalyst (Premetek, USA) was used as the control.

### Catalyst Preparation

MCL‐coated Pt nanoparticles on a carbon support, denoted as Pt@MCL (30 wt. % metal loading), were prepared by thermal decomposition reaction followed by high‐temperature heat treatment. First, 0.1 g of carbon black was dispersed in a mixture containing 5 mL of oleylamine and 127 mL of 1‐octadecene in a three‐necked round‐bottom flask using ultrasonication for 20 min to obtain a homogeneous suspension. In a separate vial, 0.09 g of Pt(acac)_2_ was dispersed in a solution containing 5 mL of oleylamine and 33 mL of 1‐octadecene, and stirred for 20 min until the solution became transparent. The resulting solution was sonicated for 5 min and heated at 120 °C for 1 h in an Ar‐saturated atmosphere to eliminate H_2_O impurities. After 1 h, the solution temperature was raised to 300 °C and maintained for 2 h to pyrolyze the metal precursors. After the reaction was complete, the solution was cooled to 80 °C. The catalyst powders were isolated by filtration and washed sequentially with copious amounts of n‐hexane and ethanol solutions. Finally, the as‐prepared catalysts were dried in a vacuum oven at 60 °C, after that the catalyst powder was heat‐treated at 800 °C for 1 h in an Ar atmosphere to form the MCL on the surface of the Pt nanoparticles. Here, oleylamine simply acts as a surfactant and is not used as an additional carbon source.^[^ ^79^ ^]^

### Electrochemical Measurements in Half‐Cell

All electrochemical measurements were performed in a standard three‐compartment electrochemical cell with a RDE, Pt wire, and an Ag/AgCl electrode acting as the working electrode, counter electrode, and reference electrode, respectively. All potential values were represented by a reversible hydrogen electrode (RHE), and catalyst inks were prepared by mixing a Pt catalyst with Nafion solution and 2‐propanol. A drop of the catalyst ink was placed on the RDE and dried at room temperature, yielding a total metal loading of ≈45 µg cm^−2^ for Pt@MCL and Pt/C catalysts. The CVs were scanned in the potential range of 0.05–1.05 V at 20 mV s^−1^ in an Ar‐saturated 0.1 m HClO_4_. The ORR polarization curves were obtained in O_2_‐saturated 0.1 m HClO_4_ at a scan rate of 5 mV s^−1^ and a rotation speed of 1600 rpm at potential between 0.05 and 1.05 V. The HOR performance was measured in H_2_‐saturated 0.1 m HClO_4_ at a scan rate of 3 mV s^−1^ and rotation speed of 1600 rpm at potentials between 0.05 and 1.0 V. Moreover, CO stripping tests were performed to quantitatively determine the EMSA of the catalysts by integrating the CO oxidation currents. While applying 0.05 V to the working electrode, 99.95 % CO gas was initially supplied to 0.1 m HClO_4_ for 15 min to facilitate CO adsorption on the surface of the Pt nanoparticles. Thereafter, the residual CO was removed from the electrolyte by bubbling Ar gas for 15 min. The CO stripping was conducted at a scan rate of 20 mV s^−1^ at potentials between 0.05 and 1.05 V. For the ADT of Pt@MCL catalyst, 10000 potential cycles were performed at 100 mV s^−1^ between 0.6 and 1.1 V in Ar‐saturated 0.1 m HClO_4_ solution.

### Preparation of MEAs with Conventional Pt/C and Pt@MCL as Anode

To construct the CL, commercial 20 wt. % Pt/C and the as‐prepared Pt@MCL catalyst were dispersed in a mixed solution of Nafion ionomer, isopropyl alcohol, deionized water, and dipropylene glycol with the ionomer‐to‐carbon ratio maintained at 0.8. For uniformly dispersed catalyst slurry, mechanical ball‐milling, and ultrasonication processes were performed. The slurry was bar‐coated on a polyimide film and completely dried for removing the residual solvent. Subsequently, both cathode and anode CLs were transferred to the NRE 211 membrane by hot‐pressing method at 140 °C for 10 min under 10 MPa. All the MEAs comprised the same cathode CLs containing commercial 20 wt. % Pt/C with a Pt loading amount of 0.2 mg cm^−2^. However, various anodes containing commercial 20 wt. % Pt/C and Pt@MCL were evaluated in this study, with the same Pt loading amount of 0.05 mg cm^−2^. The constructed anodes with reference Pt/C and Pt@MCL are depicted in Figure S9 (Supporting Information), wherein the active area of all the samples was maintained at 5 cm^2^.

### Analysis of Single‐Cell Performance

The single cells were constructed with prepared MEAs and additional fuel cell components of GDLs, Teflon gaskets, and graphite plates with a serpentine channel of 1 mm in width and height. All the components were tightly assembled by fastening 8 screws with a torque of ≈9 N m. A PEMFC test station (CNL Energy, Korea) and a potentiostat (HCP‐803, BioLogic, and France) were used for performance tests and electrochemical impedance spectroscopy (EIS), respectively. The *I–V* polarization curves were obtained using the current‐sweep method with a scan rate of 50 mA cm^−2^ under the conditions of cell temperature of 70 °C with supplying fully humidified H_2_ (300 sccm) and air (1000 sccm) without additional back‐pressure. The corresponding EIS was conducted at 0.8 V with an alternating current amplitude of 10 mV under a frequency range of 0.1 to 100 kHz. The CV was conducted with a sweeping range of 0.05–1.2 V and a scan rate of 50 mV by supplying fully humidified H_2_ (50 sccm) and N_2_ (200 sccm) to the anode and cathode, respectively.

### Anode Durability Test under Accelerated Fuel Starvation Condition

Durability tests under accelerated FS conditions were performed according to the following procedure. First, the normal operating condition was maintained by supplying fully humidified H_2_/air with a flow rate of 100 sccm at a constant current density of 0.2 A cm^−2^ and cell temperature of 70 °C. Subsequently, the anode gas was altered from H_2_ to N_2_ to simulate FS at the anode. The reverse potential appeared after ≈600 s (duration of H_2_ depletion in the single‐cell) and the MEAs with conventional Pt/C and Pt@CL were exposed to the reverse potential for 120 s.^[^ ^29^, ^51^ ^]^ Thereafter, the anode gas was reverted to H_2_, and the cell potential was recorded for 10 min under the same operating condition. The *I–V* polarization curves and the anode CVs before and after the FS protocol were obtained.

### Cathode Durability Test under Accelerated Shut‐Down/Start‐Up Condition

The SD/SU protocol comprised the following four steps as a single cycle: (1) Fuel cell operation (2) Shut‐down (3) Air/air soak, and (4) Start‐up. First, during the FC operation step, the system was operated at 0.4 A cm^−2^ for 60 s to reach a steady state. Subsequently, the shut‐down step, where the gas supply, and load were stopped, was maintained for 10 s. Following this, air gas was supplied to the anode for 60 s to form the H_2_/O_2_ boundary. To return to the start‐up step, the gas supplied to the anode was switched to hydrogen, and a load of 0.4 cm^−2^ applied for 10 s.

### MEA Chemical Durability Test under Accelerated OCV Holding Condition

An accelerated OCV holding test was conducted for 120 h by supplying partially humidified H_2_ and O_2_ with the same flow rate of 300 sccm at 90 °C under RH 30 % condition.^[^ ^34^, ^64^, ^76^ ^]^ After the OCV holding test, the drain water from the anode and cathode was collected to analyze the concentration of the total eluted fluorine ions. The *I–V* polarization curves, EIS, and linear sweep voltammetry (LSV) were measured before and after the protocol. In particular, the LSV curves were recorded at 0.4 V under the condition of supplying fully humidified H_2_ (200 sccm) and N_2_ (cathode, 200 sccm) to the anode (pseudo reference and counter electrode) and cathode (working electrode), respectively, to extract the hydrogen current density.

### Physical Characterization

TEM (JEOL 2010 FasTEM microscope) was used to determine the particle size and distribution fo the Pt nanoparticles encapsulated in MCL. Furthermore, field‐emission (FE)‐SEM (JEOL, Japan) was used to obtain the surface and cross‐sectional images of the MEAs. The fluoride‐ion concentrations eluted from the test specimens during the OCV tests were determined using a calibrated fluoride ion‐selective electrode (A214, Thermo Fisher Scientific) by mixing drain water with the same volume of the total ionic strength adjustment buffer solution.

## Conflict of Interest

The authors declare no conflict of interest.

## Supporting information

Supporting Information

## Data Availability

The data that support the findings of this study are available from the corresponding author upon reasonable request.
