# Supplementary material for: Redesign of Anode Catalyst for Sustainable Survival of Fuel Cells
Source: Adv Sci (Weinh). 2024 Jan 15;11(12):2307073. doi: 10.1002/advs.202307073 (PMC10966514; doi:10.1002/advs.202307073)
Supplement: Supplementary file 1 — Supporting Information [file ADVS-11-2307073-s001.pdf]

ADVANCED SCIENCE
Open Access

Supporting Information

for *Adv. Sci.*, DOI 10.1002/advs.202307073

Redesign of Anode Catalyst for Sustainable Survival of Fuel Cells

Keonwoo Ko, Dongsu Kim, Jiho Min, Bathinapatla Sravani, Yunjin Kim, Sanghyeok Lee, Taejun Sul, Segeun Jang and Namgee Jung**

Supporting Information

Redesign of Anode Catalyst for Sustainable Survival of Fuel Cells

*Keonwoo Ko[‡1], Dongsu Kim[‡2], Jiho Min[‡1,3], Bathinapatla Sravani[1], Yunjin Kim[1], Sanghyeok Lee[2], Taejun Sul[2], Segeun Jang[*2], Namgee Jung[*1]*

[1]Graduate School of Energy Science and Technology (GEST), Chungnam National University, 99 Daehak-ro, Yuseong-gu, Daejeon 34134, Republic of Korea.
[2]School of Mechanical Engineering, Kookmin University, Seoul 02707, Republic of Korea.
[3]Korea Institute of Energy Research (KIER), 152 Gajeong-ro, Yuseong-gu, Daejeon 34129, Republic of Korea.
E-mail: njung@cnu.ac.kr, sjang@kookmin.ac.kr

[‡] These authors have contributed equally

Contents

Figure S1. ---3

Figure S2. ---4

Figure S3. ---5

Figure S4. ---6

Figure S5. --- 7

Figure S6. --- 8

Figure S7. --- 9

Figure S8. --- 10

Figure S9. --- 11

Figure S10. --- 12

Figure S11. --- 13

Figure S12. --- 14

Figure S13. --- 15

Figure S14. --- 16

Figure S15. --- 17

Figure S16. --- 18

Table S1. ---19

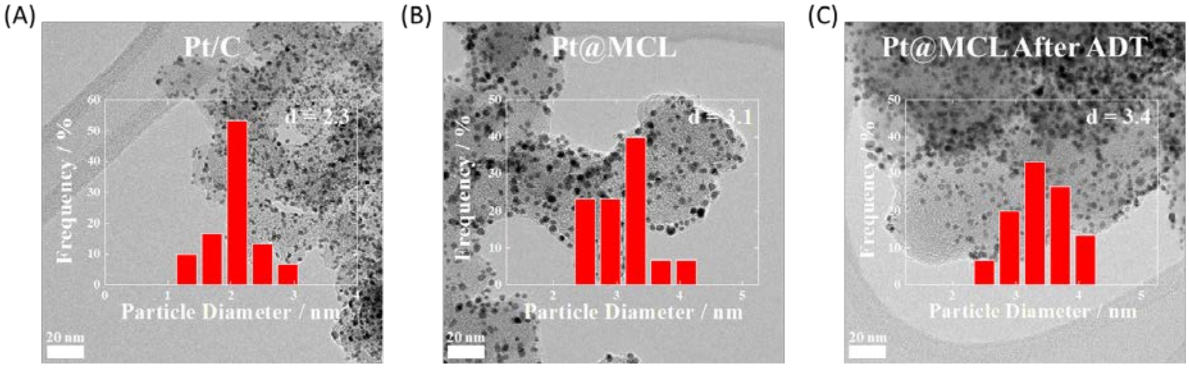

Figure S1. Low-magnification TEM images and particle size distributions of (A) Pt/C (fresh), (B) Pt@MCL (fresh), and (C) Pt@MCL after ADT.

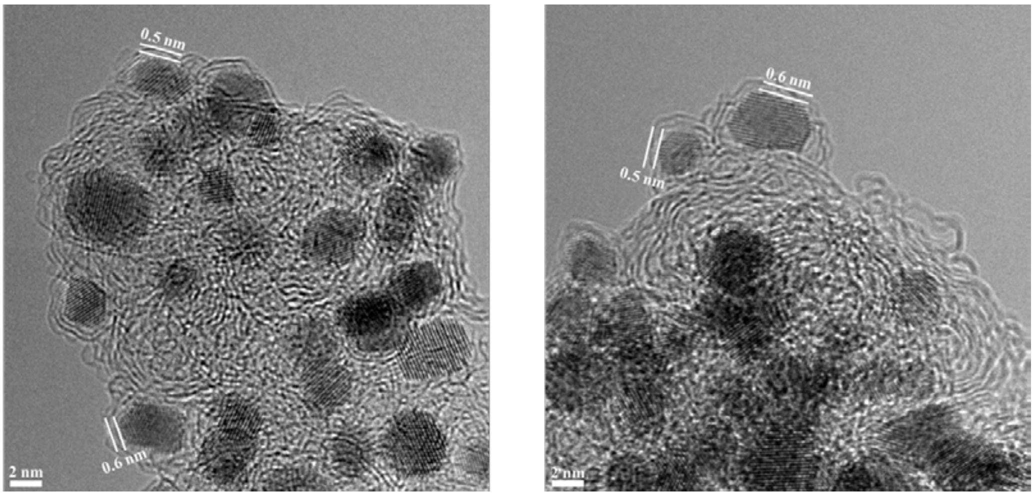

Figure S2. TEM images and carbon shell thickness of Pt@MCL.

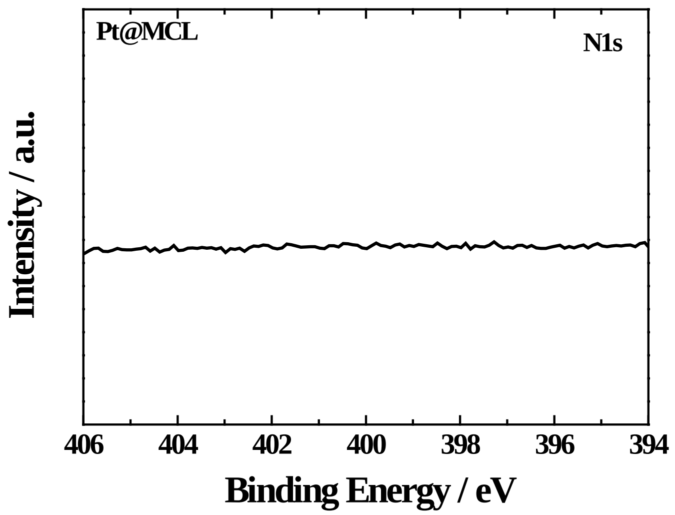

Figure S3. N1s core-level XPS spectra of Pt@MCL catalyst.

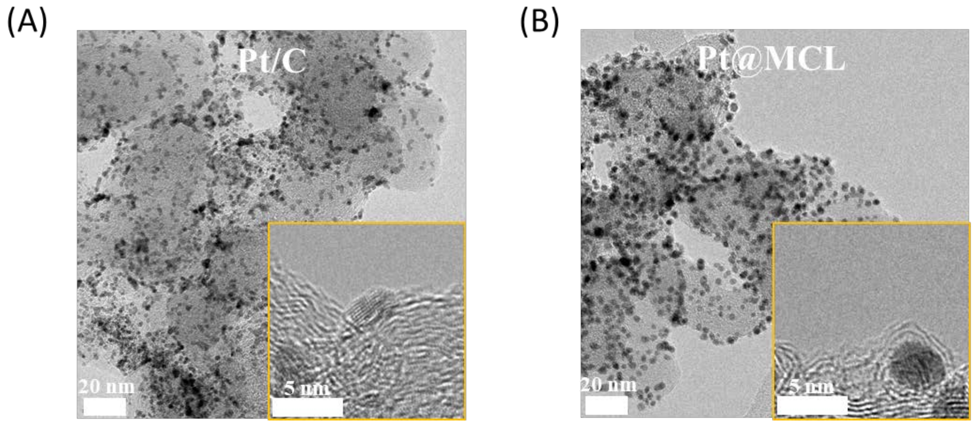

Figure S4. TEM image of (A) Pt/C and (B) Pt@MCL. The inset in each figure shows the HR-TEM image of a Pt nanoparticle in the corresponding figures.

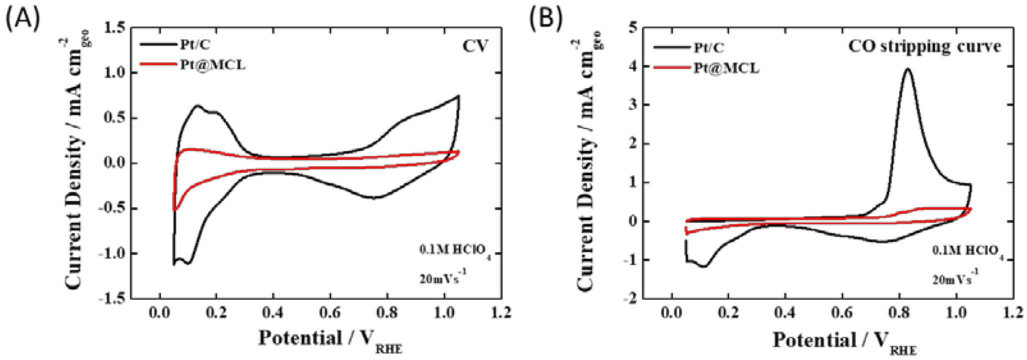

Figure S5. (A) CVs and (B) CO stripping curves of the Pt/C and Pt@MCL catalysts.

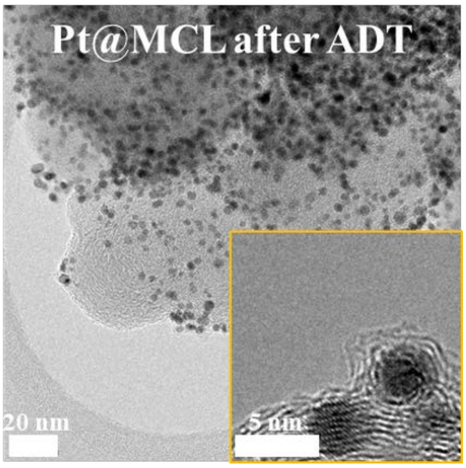

Figure S6. TEM image of Pt@MCL after ADT. The inset shows the HR-TEM image of the Pt nanoparticles.

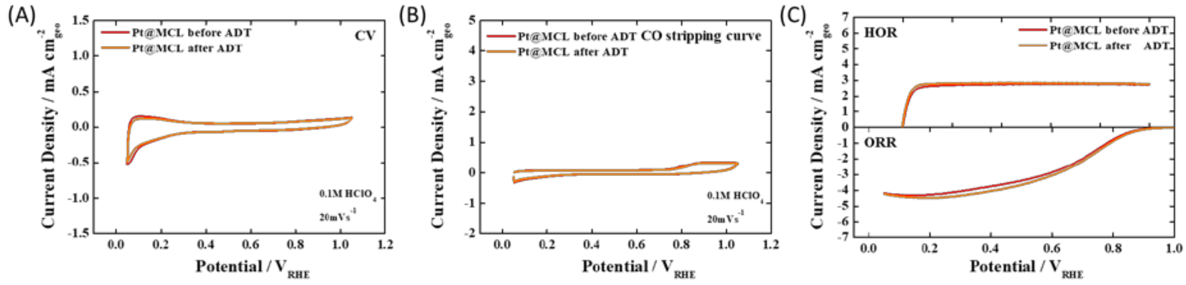

Figure S7. Electrochemical properties of the Pt@MCL catalyst before and after ADT. (A) CVs, (B) CO stripping curves, and (C) HOR and ORR polarization curves.

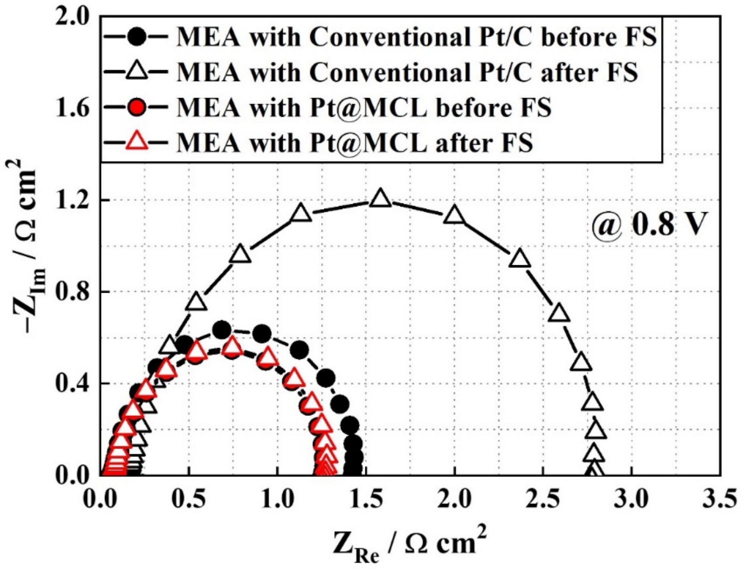

Figure S8. EIS spectra at 0.8 V of MEAs before and after the simulated FS tests.

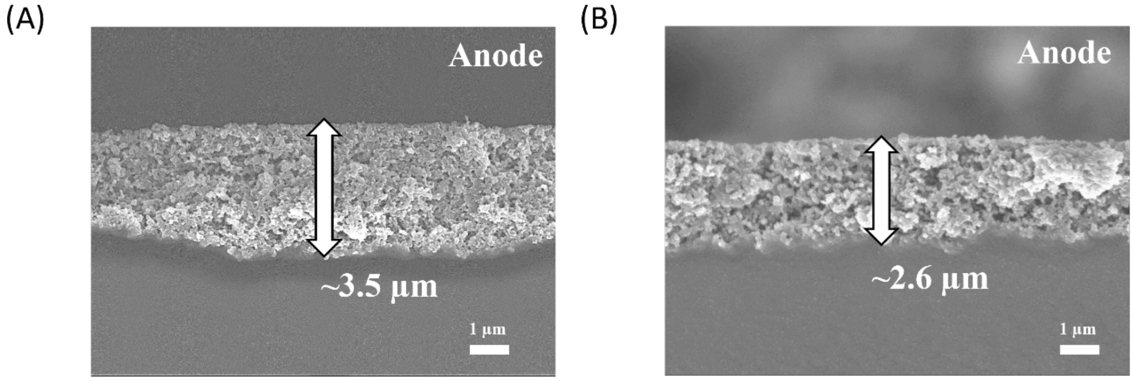

Figure S9. Cross-sectional SEM images of (A) MEA with conventional Pt/C and (B) MEA with Pt@MCL before FS tests.

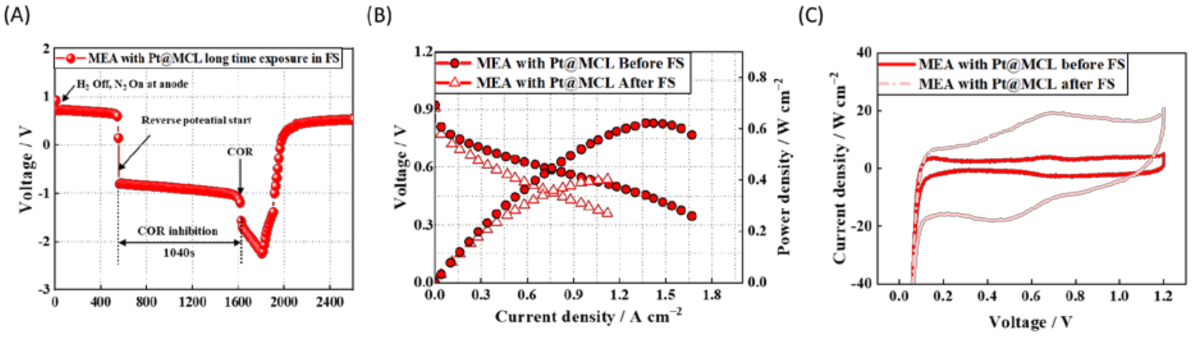

Figure S10. (A) Instantaneous cell voltage behavior of MEA with Pt@MCL during a longer FS test. (B) Polarization curves and (C) CVs of the MEA before and after the FS test.

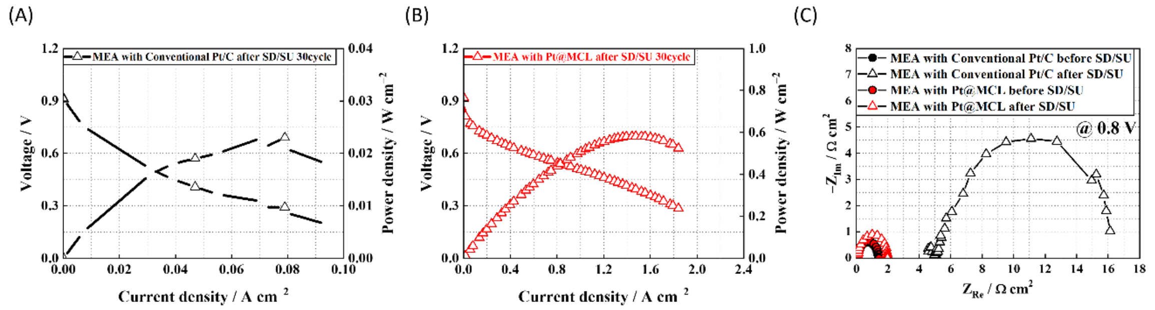

Figure S11. EIS spectra at 0.8 V of the MEAs before and after SD/SU tests.

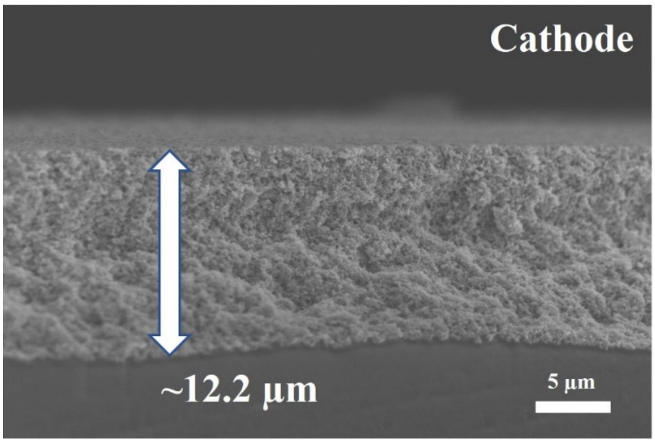

Figure S12. Cross-sectional SEM images of the conventional MEA before the SD/SU test.

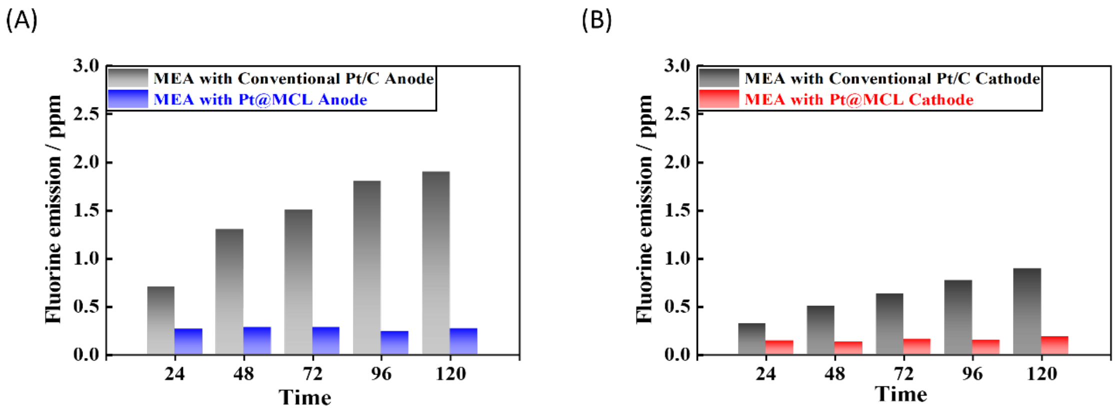

Figure S13. (A) Anode fluoride-ion emission during OCV holding test every 24h. (B) Cathode fluoride-ion emission during OCV holding test every 24h.

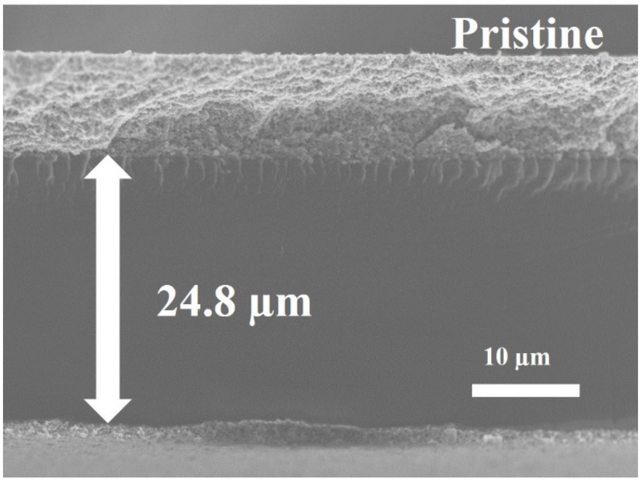

Figure S14. Cross-sectional SEM images of the conventional MEA before the OCV holding test

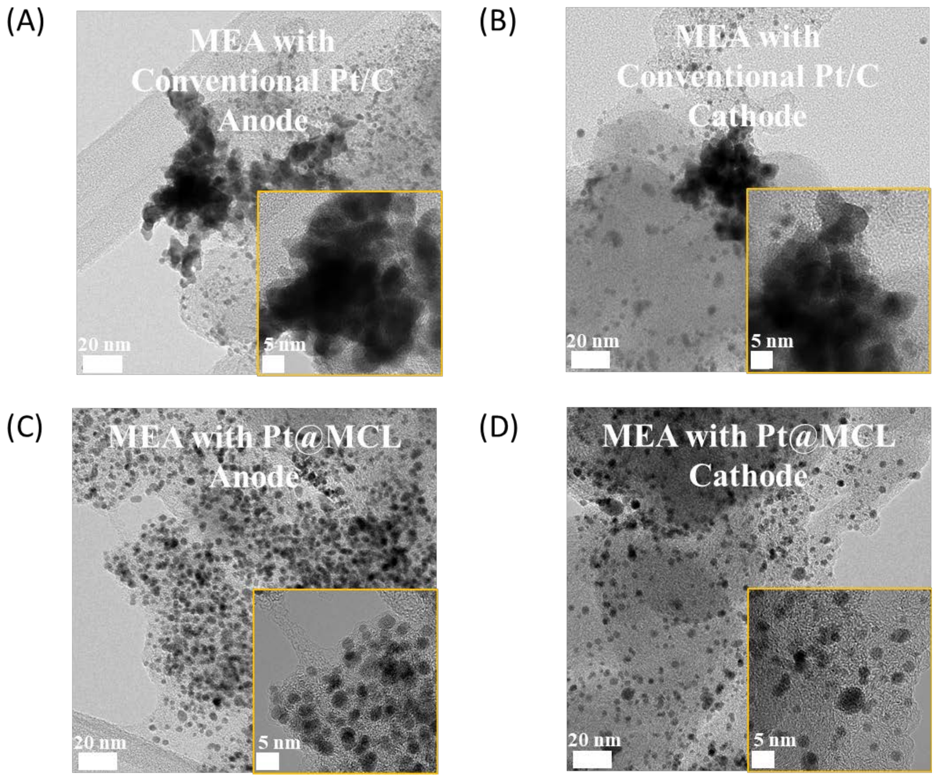

Figure S15. TEM images of the catalysts after OCV holding test. (A) Anode catalysts (Pt/C) and (B) cathode catalysts (Pt/C) of the conventional MEA, and (C) anode catalysts (Pt@MCL) and (D) cathode catalysts (Pt/C) of the MEA with Pt@MCL.

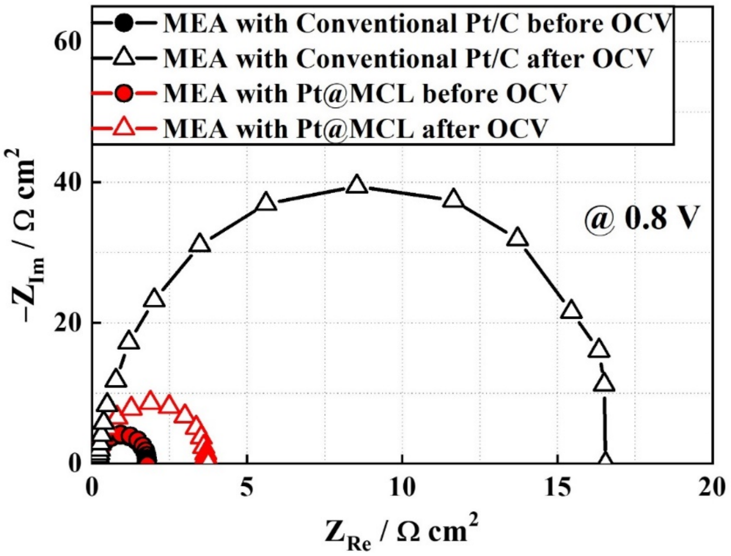

Figure S16. EIS spectra at 0.8 V of the MEAs before and after OCV holding tests.

Table S1. Detailed durability effects of Pt@MCL at the anode in the MEA.

**Fuel starvation**	Before AST<br>peak power density [W cm^{-2}]	After AST<br>peak power density [W cm^{-2}]
**MEA with Conventional Pt/C**	0.687	0.449
**MEA with Pt@MCL**	0.703	0.687
**Durability<br>(Anode CL)**	**MEA with Conventional Pt/C<br>degradation rate<br>- 34.65%**	**MEA with Pt@MCL<br>degradation rate<br>- 2.28%**

**Shut-down / Start-up**	Before AST<br>peak power density [W cm^{-2}]	After AST<br>peak power density [W cm^{-2}]
**MEA with Conventional Pt/C**	0.674	0.023
**MEA with Pt@MCL**	0.667	0.582
**Durability<br>(Cathode CL)**	**MEA with Conventional Pt/C<br>degradation rate<br>- 96.59%**	**MEA with Pt@MCL<br>degradation rate<br>- 12.75%**

**OCV holding test**	Before AST<br>peak power density [W cm^{-2}]	After AST<br>peak power density [W cm^{-2}]
**MEA with Conventional Pt/C**	0.704	0.455
**MEA with Pt@MCL**	0.693	0.575
**Durability<br>(Membrane)**	**MEA with Conventional Pt/C<br>degradation rate<br>- 35.37%**	**MEA with Pt@MCL<br>degradation rate<br>-17.03%**
